# The genetic basis of onset age in schizophrenia: evidence and models

**DOI:** 10.3389/fgene.2023.1163361

**Published:** 2023-06-27

**Authors:** Na Zhan, Pak C. Sham, Hon-Cheong So, Simon S. Y. Lui

**Affiliations:** ^1^Department of Psychiatry, School of Clinical Medicine, The University of Hong Kong, Hong Kong, Hong Kong SAR, China; ^2^ Centre of PanorOmic Sciences, The University of Hong Kong, Hong Kong, Hong Kong SAR, China; ^3^ State Key Laboratory of Brain and Cognitive Sciences, The University of Hong Kong, Hong Kong, Hong Kong SAR, China; ^4^ School of Biomedical Sciences, The Chinese University of Hong Kong, Shatin, Hong Kong SAR, China; ^5^KIZ-CUHK Joint Laboratory of Bioresources and Molecular Research of Common Diseases, Kunming Institute of Zoology and the Chinese University of Hong Kong, Shatin, Hong Kong SAR, China; ^6^ Department of Psychiatry, The Chinese University of Hong Kong, Shatin, Hong Kong SAR, China; ^7^ CUHK Shenzhen Research Institute, Shenzhen, China; ^8^ Margaret K. L. Cheung Research Centre for Management of Parkinsonism, The Chinese University of Hong Kong, Shatin, Hong Kong SAR, China; ^9^ Brain and Mind Institute, The Chinese University of Hong Kong, Shatin, Hong Kong SAR, China; ^10^ Hong Kong Branch of the Chinese Academy of Sciences Center for Excellence in Animal Evolution and Genetics, The Chinese University of Hong Kong, Shatin, Hong Kong SAR, China

**Keywords:** schizophrenia, onset age, subtyping, susceptibility genes, modifier genes

## Abstract

Schizophrenia is a heritable neurocognitive disorder affecting about 1% of the population, and usually has an onset age at around 21–25 in males and 25–30 in females. Recent advances in genetics have helped to identify many common and rare variants for the liability to schizophrenia. Earlier evidence appeared to suggest that younger onset age is associated with higher genetic liability to schizophrenia. Clinical longitudinal research also found that early and very-early onset schizophrenia are associated with poor clinical, neurocognitive, and functional profiles. A recent study reported a heritability of 0.33 for schizophrenia onset age, but the genetic basis of this trait in schizophrenia remains elusive. In the pre-Genome-Wide Association Study (GWAS) era, genetic loci found to be associated with onset age were seldom replicated. In the post-Genome-Wide Association Study era, new conceptual frameworks are needed to clarify the role of onset age in genetic research in schizophrenia, and to identify its genetic basis. In this review, we first discussed the potential of onset age as a characterizing/subtyping feature for psychosis, and as an important phenotypic dimension of schizophrenia. Second, we reviewed the methods, samples, findings and limitations of previous genetic research on onset age in schizophrenia. Third, we discussed a potential conceptual framework for studying the genetic basis of onset age, as well as the concepts of susceptibility, modifier, and “mixed” genes. Fourth, we discussed the limitations of this review. Lastly, we discussed the potential clinical implications for genetic research of onset age of schizophrenia, and how future research can unveil the potential mechanisms for this trait.

## Introduction

Schizophrenia is a severe psychiatric disorder, influenced by multiple factors, with an estimated age-standardized point prevalence of around 0.28% worldwide (Charlson et al., 2018). Early epidemiological studies suggested that schizophrenia aggregates in families, implicating its heritability and the important role of genetics in the aetiology of schizophrenia (Frangos et al., 1985; Kendler et al., 1985). Previous studies suggested that the heritability of schizophrenia is high (i.e., around 60%–80%) (Hilker et al., 2018). Many linkage studies were conducted in high-risk families in the “pre-Genome-Wide Association Study (GWAS) era” (Coon et al., 1993; Chowdari et al., 2002). To date, the genome-wide approach has become widely available and inexpensive, and a growing number of genome-wide association studies (GWAS) have been applied to schizophrenia samples. According to the latest GWAS meta-analysis conducted by the Psychiatric Genomics Consortium (PGC), nearly 300 single nucleotide polymorphisms (SNPs) have been identified as susceptibility variants for schizophrenia (Trubetskoy et al., 2022). Apart from the common variants found in the PGC meta-analysis, rare variants like CNV and *de novo* variants have also been found to influence the risk of developing schizophrenia (Girard et al., 2011; Rees et al., 2020). Besides genetic factors, environmental factors such as cannabis use, childhood trauma, and migration can also increase the risk of developing schizophrenia (Brown, 2011; Stepniak et al., 2014). Although many risk factors have been identified, there is still a long way to go before we may fully translate these findings to improve the diagnosis and treatments of schizophrenia. For instance, although a lot of common variants were found to be related to liability to schizophrenia, each of them only accounts for a very small effect. This issue may be related to genetic heterogeneity and phenotypic complexity of this disorder. One strategy to overcome such problems is trying to dissect schizophrenia into more homogeneous subtypes, such as deficit syndrome of schizophrenia (Carpenter et al., 1988), treatment-resistant schizophrenia (Howes et al., 2017) and early-onset schizophrenia (Remschmidt and Theisen, 2012).

Age at onset (AAO) of psychiatric disorders has received much attention in the past several decades (Solmi et al., 2021). Schizophrenia patients with early AAO showed higher severity of clinical symptoms and poorer prognosis (Immonen et al., 2017). One commonly-used definition of AAO is the age when the first psychotic symptom emerges (Remschmidt and Theisen, 2012). However, two other possible definitions of AAO have been proposed (De Girolamo et al., 2019). Specifically, AAO can also refer to the age when the first “morpho-functional pathological process” appears. For instance, aberrant brain network and grey matter loss have been reported in schizophrenia patients, and these pathological changes may happen before the observable clinical symptoms become detectable (McCutcheon et al., 2020). Alternatively, AAO can be defined as the age when the first “observable” sign of psychosis appears (such as onset of frank psychotic symptoms requiring psychiatric treatment). Although some studies have investigated AAO in psychiatric disorders, evidence at the genetic level remains limited and inconsistent.

Elucidating the genetic basis for AAO in schizophrenia has theoretical and clinical implications. The genetic basis for AAO may reflect the biology of schizophrenia and pave ways for future prediction of schizophrenia onset, and thus may facilitate timely intervention and prevention (Correll et al., 2018; McGorry and Mei, 2018). Research on AAO can also help in subtyping schizophrenia (Yin et al., 2018; Yin et al., 2019). In this article, we reviewed the current genetic evidence for AAO in schizophrenia, and discussed potential conceptual frameworks for the genetic architecture of AAO, which could be useful in addressing the previous limitations and directing future research.

## Methods

Regarding the search strategy, we first conducted a search in the PubMed, Medline, and PsycInfo databases using the following search terms: [(“schizophrenia” OR “schizophrenic”) AND (“age of onset” OR “age at onset” OR “onset age” OR “early onset” OR “late onset”) AND (“gene” OR “genes” OR “genetic” OR “genetics” OR “locus” OR “loci” OR “SNP”)]. The search was limited to English language peer-reviewed articles, published in the period from January 1970 to April 2023. We only considered studies using human subjects. Then, we manually searched references of the retrieved articles for additional relevant studies.

For study selection, we included family and twin studies, as well as studies on common variants and rare variants (i.e., linkage, candidate gene association, genome-wide association, copy number variation, and exome sequencing studies) which examined the genetic contribution to AAO in schizophrenia patients. In the literature, variables representing AAO as defined by various criteria were considered, including the age when the first psychotic symptom emerged, the first contact with mental health service for psychosis, or the time when first diagnosis of psychosis was made. The retrieved articles were reviewed by ZN, and checked by LSSY.

### Current evidence on the genetic basis of age at onset in schizophrenia

In the retrieved studies, the sample size ranged widely. Moreover, only a minority of retrieved studies considered gender stratification using subgroup analysis, and very few studies reported medication prescription status. Schizophrenia was diagnosed differently using various classification systems including the ICD-10, DSM-III, DSM-IV, and DSM-5. The majority of retrieved studies treated AAO as a continuous variable, whilst some treated AAO as a categorical variable and contrasted the subgroups with early versus late AAO. The cut-off age for early-onset and adulthood-onset schizophrenia also differed in the retrieved studies.

#### Family and twin studies

An early study on multiple-affected families (with 270 schizophrenia probands and 3,997 first- and second-degree relatives) found that the AAO in probands was positively correlated with that in offsprings (*r* = 0.42), siblings (*r* = 0.44), and nephews and nieces (*r* = 0.13) (Kendler et al., 1990). Another study pooled data from 5 proband-sibling studies on AAO, and concluded that the proband-sibling correlation of AAO ranged from 0.590 to 0.857 (Crow and Done, 1986).

The impact of familial factors on AAO in schizophrenia was also reviewed by a previous study using the data gathered from 4 twin studies, and the correlation of AAO between monozygotic (MZ) twins was found to be around 0.57 to 0.86, with a weighted mean of 0.68 (Kendler et al., 1987). A more recent study identified 788 pairs of patients with schizophrenia spectrum disorder (including 448 pairs of schizophrenia patients) in the Danish nationwide twin registry, and reported that the familial risk for schizophrenia was 4.7 times higher in twin pairs of early-onset schizophrenia (aged <22), compared with twin pairs of late-onset schizophrenia, and the hazard ratio for schizophrenia spectrum disorder was around 4.4 (Hilker et al., 2017). Moreover, they reported closer AAO in monozygotic than dizygotic twins, implicating the importance of familial and genetic influence in determining schizophrenia AAO.

The evidence for heritability of AAO is limited. One previous study recruited 717 schizophrenia patients from 327 Mexican and Central American families reported an heritability estimate of 0.33 for AAO (*p* = 0.00004), and found that gender would be a significant covariate in affecting AAO (Hare et al., 2010).

Some limitations were notable in these family and twin studies. For instance, such methods relied on the co-occurrence of the phenotypes of interest (schizophrenia, AAO) to estimate genetic contributions, but they could not identify specific genetic variants or genes contributing to the outcome. Moreover, case status in the members of multiple-affected families may change with time, and therefore the future risk of developing late-onset schizophrenia in some currently unaffected members may not be accounted for (Kendler et al., 1987). Third, early detection and thus early AAO may be more likely in the second twin who developed schizophrenia, because of increased mental health literacy among the families who had their first twin diagnosed with the same illness (Kendler et al., 1987; Hilker et al., 2017).

### Common genetic variants

#### Genetic linkage studies

Linkage analysis is known as the first DNA-based method which can unbiasedly identify genomic regions among high-risk families without any preconception about the role of certain genes (Henriksen et al., 2017). A previous study using this approach identified six markers for AAO with the maximum-likelihood LOD score that reached the suggestive linkage criteria among 94 affected sibling pairs, but none of them achieved genome-wide statistical significance (Cardno et al., 2001). One of the top markers with LOD of 2.54 (D17S787, genome-wide *p* = 0.27) was on chromosome 17, and another one (D13S158, on chromosome 13) with LOD of 1.68 was reported to coincide with a previously reported linkage region for schizophrenia (Pulver et al., 1998). A linkage study (Mowry et al., 2004) searched for susceptibility loci on chromosome 22q among a multi-centered sample with 779 schizophrenia pedigrees. Although previous research reported some evidence for the association of chromosome 22q with schizophrenia, this study did not find any positive associations of this region with either schizophrenia or AAO (Mowry et al., 2004). Another linkage study (Fanous et al., 2007) conducted a genome-wide scan among 270 Irish families, and explored “modifier loci” for multiple phenotypes in schizophrenia. The findings suggested a peak association of AAO with the region of 6q23.1-6q25.2 (D6S1040-D6S2420, with LOD of 2.26), but it did not surpass the threshold of genome-wide significant linkage (Fanous et al., 2007). A more recent study also conducted a genome-wide scan of AAO among 295 families with schizophrenia patients and first-degree relatives, by using the ordered subset linkage analysis. They identified a significant linkage evidence for the association of 2q22.1 with younger AAO, with LOD scores of 4.17 (*p* = 0.001) (Lien et al., 2011). Interestingly, a loss at 2q22.1 nearby the *THSD7B* gene was reported in an unaffected twin from a copy number variation study of schizophrenia (Castellani et al., 2014).

Among the aforementioned studies, only the most recent one (Lien et al., 2011) reported significant linkage findings for AAO. Compared to the other studies (Cardno et al., 2001; Mowry et al., 2004; Fanous et al., 2007), this study (Lien et al., 2011) did not stratify subjects using a simple cut-off age for AAO, but used a new method to select subsets with varied AAO by ranking this trait. The usage of a more advanced statistical method and its larger sample size may explain the significant findings, whereas previous studies may be limited by their smaller sample size (Cardno et al., 2001) and lower number of candidate markers (e.g., one of them only genotyped 10 microsatellite markers (Mowry et al., 2004)). Moreover, some studies combined the samples of multiple-affected families from different recruitment sites; the resultant ethnic heterogeneity would be a concern (Mowry et al., 2004). Although conducting genome-wide scans would allow for the search of more risk regions, the issue of multiple testing adjustments can be a limitation.

#### Candidate-gene association studies

Compared with linkage analysis, the candidate-gene approach can detect variants with small effects but does not require large families of affected and unaffected members as study samples. Candidate genes could be selected based on previous linkage studies or biological assumptions of possible relationships with schizophrenia or AAO. Dysregulation in the dopaminergic system is a widely-accepted model for the pathophysiology of schizophrenia (Davis et al., 1991; Hietala and Syvälahti, 1996), with a growing number of studies showing significant associations between dopamine receptor genes (e.g., *DRD2, DRD3*) and psychosis or schizophrenia (Schizophrenia Working Group of the Psychiatric Genomics Consortium, 2014; Buck et al., 2022). Explorations on the role of these dopamine receptor genes on other phenotypic features of schizophrenia have been conducted. One previous study focused on two polymorphisms *Taq1* B1/B2 and *Taq1* A1/A2 (rs1079597 and rs1800497) on the *DRD2* gene and reported that the haplotype B2A2 was significantly associated with late-onset schizophrenia. Moreover, they found that AAO would increase with the number of this haplotype (Dubertret et al., 2001). Another study genotyped 7 SNPs on the *DRD2* gene, and found that the polymorphism rs2734839 was significantly associated with schizophrenia diagnosis and AAO, and schizophrenia patients carrying the GA genotype showed a later AAO than those carrying the AA genotype (*p* = 0.012) (Voisey et al., 2012). Another study compared childhood-onset schizophrenia (COS: AAO ranged from 3 to 16) and adult-onset schizophrenia (AOS: AAO ranged from 18 to 25) patients, and found that the frequency of *DRD2* genotypes significantly differed in these two groups, with a lower frequency of risk allele C of SNP rs2514218 in the COS group (Alfimova et al., 2023). Several other studies also tested SNPs in *DRD2*, but did not report any positive findings (Itokawa et al., 1993; Xiao et al., 2013; Michalczyk et al., 2020a). Among them, one study genotyped the SNP rs1800497, but did not detect any significant association with AAO (Michalczyk et al., 2020b); two studies investigated the SNP rs1799732, and both reported negative findings for its association with AAO (Xiao et al., 2013; Michalczyk et al., 2020b). For the *DRD3* gene, one study reported that the *Bal* I allele 2 was associated with earlier AAO, and such association was stronger in male than female subjects (Griffon et al., 1996). However, another study did not find any association between AAO and *Bal* I polymorphism (Inada et al., 1995). One study genotyped 10 SNPs in the *DRD3* gene, and found three SNPs (rs905568, rs7611535 and rs6762200) associated with AAO in male subjects only (Renou et al., 2007). One study reported that the Ser9Gly (−) genotype was associated with higher AAO in female subjects only (Godlewska et al., 2010), but another study did not find any difference when they stratified the AAO into categorical variables and compared the distribution of the same genotype between early-onset (aged <25) and late-onset (aged ≥25) schizophrenia (Fathalli et al., 2008).

The Catechol-O-methyltransferase (*COMT*) gene, located on chromosome 22q11.2, is another well-known candidate gene for schizophrenia, involved in dopamine and norepinephrine metabolisms. Some studies tested the association of the *COMT* gene with AAO in schizophrenia, but the results were not consistent. Specifically, one study reported that the functional polymorphism Val108/158 Met did not show any significant relationship with either disease susceptibility or AAO. However, the dose effect of Met66 in the *BDNF*, another candidate gene for schizophrenia, was found to be weakly but significantly associated with AAO (*r* = 0.162, *p* = 0.042) (Numata et al., 2007). Other studies have examined the effects of the *COMT* polymorphism Val158Met on AAO, but most of them reported negative findings (Nieratschker et al., 2010; Pelayo-Terán et al., 2010; Tylec et al., 2017). However, one study recruited 80 patients with schizophrenia-spectrum disorders, and reported that the Val/Val genotype was associated with earlier AAO (Estrada et al., 2011). For the *BDNF* gene, one study reported a significant association between the *BDNF* Val66Met and AAO (Mané et al., 2017).

The *NRG1* gene plays an important role in the nervous system and is related to neuronal connectivity (Lajtha et al., 2009). Since the *NRG1* was first reported as a susceptible gene for schizophrenia (Stefansson et al., 2002), the relationship of the *NRG1* with other phenotypes including AAO had been studied. Several studies did not find that AAO was associated with the polymorphisms Arg38Gln (rs3924999), rs6994992 and SNP8NRG221132 (Hong et al., 2004; Crowley et al., 2008; Voineskos et al., 2009). However, a phenotype-based genetic association study found that several haplotypic variants (i.e., SNP8NRG221533, SNP8NRG241930 and SNP8NRG243177) in the *NRG1* gene were under-represented in schizophrenia patients compared with healthy people, suggesting protective effects. Two of these variants (i.e., SNP8NRG221533 and SNP8NRG243177) were also under-represented in schizophrenia patients with early AAO (aged <20) (Papiol et al., 2011). Moreover, another study reported that AAO in schizophrenia may be associated with HapICE risk haplotype (which contained the aforementioned variants of the *NRG1* gene), and increased Type III mRNA expression was involved (Weickert et al., 2012). Another study using a Japanese sample replicated the finding that increased Type III expression was associated with early AAO in schzophrenia (Yoshimi et al., 2016).

An early study on Caucasian and African American samples examined the role of Apolipoprotein E (*ApoE*) in schizophrenia. Although they did not find any significant difference between schizophrenia patients and controls, the *ApoE* ε4 genotype was found to be associated with an earlier AAO in schizophrenia patients (Arnold et al., 1997). Similar findings were reported in other studies (Durany et al., 2000; Kampman et al., 2004; Akanji et al., 2009). By contrast, two other groups of researchers investigated the *ApoE* genotype in Spanish and Italian populations, but failed to replicate the associations of *ApoE* genotype with either the case/control status or AAO (Sorbi et al., 1998; Sáiz et al., 2002).

The immune system plays an important role in neurodevelopmental disorders and schizophrenia (Horváth and Mirnics, 2014; Meltzer and Van de Water, 2017), and genetic variants related to immune functioning have been implicated in schizophrenia and related disorders (Michel et al., 2012). For example, one study investigated whether the polymorphism of *TNF-RII* (the receptor of cytokine TNFα) was associated with schizophrenia and brain morphology. The results showed that the *TNF-RII* polymorphism was not associated with schizophrenia status; but individuals who carried homozygotes allele 1 were found to have earlier AAO compared to individuals who carried 1 or 2 copies of allele 2 (Wassink et al., 2000).

The syndrome of 22q11 deletion is associated with a very strong risk for developing schizophrenia. Around 25% of individuals with 22q11 deletion syndrome will develop schizophrenia. One study genotyped 5 polymorphisms within the 22q11 deletion region, and found that the CC genotype of the *ZNF74* gene 1150T/C polymorphism was associated with earlier AAO in schizophrenia (Takase et al., 2001). Moreover, because the polymorphism was not associated with schizophrenia status, it has been argued that the *ZNF* polymorphisms might be a “gene modifier” that affects AAO only, rather than influencing the risk of developing schizophrenia.

Other markers like the Epidermal growth factor (*EGF*) (Lee et al., 2006), *MTHFR* (Vares et al., 2010), *RELN* (Wedenoja et al., 2010), the lysine 9 of histone 3 (*H3K9me2*) (Gavin et al., 2009), *DBH* (Barlas et al., 2012), and *ANKK1* (Zhang et al., 2014) have also been examined. Most of these variants were found to be associated with AAO in schizophrenia, but not with the disease status of schizophrenia. Specifically, variants in *EGF* was reported to be significantly associated with AAO in male subjects only (Lee et al., 2006).

Although the candidate-gene association approach can only detect a limited number of variants at a time and requires *a priori* knowledge regarding biologically plausible assumptions, it remains popular because of its simplicity in study design and analysis. However, as shown in our review, previous studies using the candidate-gene approach reported inconsistent results, for several possible reasons. Firstly, some studies may be susceptible to population stratification. Unlike genome-wide association studies, in which the population stratification can be controlled using principal component analysis or mixed models, it is often difficult to detect and correct for this issue in candidate-gene studies involving only a few markers. Besides, in general, candidate-gene studies employed *p* < 0.05 as the significance threshold, yet not all studies had rigorously controlled for multiple testing (e.g., if multiple SNPs and/or phenotypes were studied). These problems may result in false-positive findings (Chang et al., 2014). There may also be other “hidden” issues of multiple testing, for example, some negative findings may not be reported. In fact, a review pointed out that only around 2% of findings of studies using the candidate-gene association approach could be replicated (Hirschhorn et al., 2002). Besides, the heterogeneity in the definition of AAO in the extant literature could be an issue, lowering the comparability of findings between different studies. In fact, some studies did not even mention how the AAO was ascertained and defined (Arnold et al., 1997; Takase et al., 2001; Gavin et al., 2009). Lastly, treating AAO as a continuous or categorical variable may result in different findings. To conclude, although previous candidate gene association studies have revealed some interesting findings, the replicability of approach is generally low.

#### Genome-wide association studies (GWAS)

During the past two decades, a rapidly growing number of GWAS studies have been conducted to discover common genetic variants for complex human diseases and traits. This method can test many SNPs at the same time and do not need *a priori* candidate gene selection. However, to our knowledge, only three GWAS papers have been published on AAO in schizophrenia, with two studies based on European populations, and one on East-Asian population. The first GWAS on schizophrenia AAO was published in 2011, based on 1,162 European-American individuals (Wang et al., 2011). This study found 104 SNPs that surpassed the suggestive threshold of significance (*p* = 1 × 10^−4^), and the strongest associations were observed in SNP rs7819815 located at 8q24.22 (*p* = 3.10 × 10^−7^) which is near the gene *ZFAT*, and in SNP rs17039583 located at gene *COL25A1* (*p* = 4.30 × 10^−6^). Moreover, two regions (1q43 and 7q22.3) showed ‘gene-gender’ interaction effects in the sample, with *p* values closed to significance threshold. However, an attempt to replicate these findings using an independent sample only found nominal significance of associations for their flanking SNPs. The second GWAS study (Bergen et al., 2014) was conducted in a larger sample with 2,387 schizophrenia patients, and the authors applied linear regression models on AAO. Several SNPs with strong association signals were observed. The reported strongest association was at rs11999864 located on chromosome 9 (*p* = 1.52 × 10^−7^), which spanned two genes *OR2K2* and *KIAA0368* (Bergen et al., 2014). The authors examined the top SNPs, and found they were not related to the case-control status, suggesting that these SNPs may not influence disease susceptibility. Additionally, pathway analysis was conducted for the top SNPs, and the results indicated that the intracellular signaling pathway was significantly associated with AAO (Bergen et al., 2014). This group of researchers also attempted to test the possible association between the genetic risk burden of schizophrenia and AAO, and calculated the polygenic score based on a GWAS conducted in a Swedish sample (Bergen et al., 2012), but the results showed that the early-onset group (aged <21) did not show any increased polygenic risk burden of schizophrenia. The third GWAS study on schizophrenia AAO utilized a smaller East-Asian sample (N = 185), and identified 14 SNPs on chromosomes 1, 4, 6, 7, 18, 19 and 21 that reached the suggestive significance threshold (1 × 10^−4^) (Woolston et al., 2017). They also built a multi-SNP genetic risk score (GRS) model using the 14 SNPs as candidate loci, and found a significant trend in the risk scores across different AAO groups, with the earlier onset groups having higher GRS. However, the top loci found in this study (Woolston et al., 2017) did not overlap with the variants discovered in other GWAS for studying AAO.

GWAS is more robust in detecting true risk loci for complex disorders, although it involves heavier multiple testing burdens (Tam et al., 2019). None of the three GWAS in this review reported genome-wide significant SNPs associated with AAO, and one of the reasons would be the very stringent *p*-value threshold. Moreover, the top SNPs found in these studies showed little overlap, and this may indicate the heterogeneity in study population and methods. For instance, the first two GWAS (Wang et al., 2011; Bergen et al., 2014) utilized samples with European ancestry and treated AAO as a continuous variable, while the third study (Woolston et al., 2017) utilized an East-Asian sample and treated AAO as a categorical variable.

The divergent findings in these GWAS studies may also be attributable to the variations in defining AAO, as mentioned earlier (De Girolamo et al., 2019). In fact, the variations of AAO may be related to the cohort effect. Evidence supported the documented AAO in clinical cases with schizophrenia has becoming younger over the years (Di Maggio et al., 2001), which may be related to the improving mental health literacy, effective early detection service, and changes in how psychiatric classification systems define schizophrenia.

#### Rare genetic variants

To date, few studies on rare variants for schizophrenia AAO have been conducted. One study examined rare copy number variants (CNV) in 629 schizophrenia patients, and reported a positive correlation between the total CNV burden and AAO, yet this finding did not survive multiple testing correction (Martin et al., 2015). This preliminary finding should be verified in future research with larger samples. Another study investigated the gene (large, rare CNV)-environment (cannabis abuse) interaction effect on AAO (Martin et al., 2014), and found that large, rare deletions were associated with later schizophrenia AAO. Although patients with and without large, rare duplications did not differ in AAO, only those patients *without* large, rare duplications but abused cannabis had earlier AAO (Martin et al., 2014). In another study, micro-duplications in the *MYT1L* gene was found to be more common in childhood-onset than in adult-onset schizophrenia patients (Lee Y. et al., 2012). Loss of function (LoF) variants in the *SCHEMA* gene have been reported to affect phenotypic features of schizophrenia, such as earlier AAO and poorer treatment outcomes (Cohen et al., 2022). The previous rare-variant studies were usually limited by issues of sample size, choice of methods to improve statistical power, and functional interpretation. Having said that, rare variants are believed to be important in AAO, because they usually have larger effects than the common variants on phenotypic features, and may partly contribute to the heritability unexplained by GWAS findings (Goswami et al., 2021).

## Discussions

### Conceptual framework of the genetic architecture of AAO in schizophrenia

To uncover the genetic architecture of a phenotypic trait, our ultimate goal is to identify the number of variants that could contribute to the phenotype, the effect size and frequency of each of these genetic variants, and how the genes or variants would interact (Kopp and Hermisson, 2006; Timpson et al., 2018). For the genetic architecture of schizophrenia AAO, previous hypothesis-free studies (mainly GWAS) have found very few variants that surpassed the genome-wide threshold of significance. Moreover, the suggestive genetic variants for schizophrenia AAO showed low levels of consistency and replicability across the aforementioned studies. Moreover, research in rare variants for schizophrenia AAO has been limited.

Fanous and Kendler (2008) proposed three possible ways for genes to affect the phenotypic features of schizophrenia, i.e., 1) susceptibility genes, 2) modifier genes, and 3) susceptibility-modifier genes. Susceptibility genes are those genes that can contribute to the risk of developing the disease directly. After reviewing the previous results, we observed little overlap between the schizophrenia AAO genes identified using GWAS and the schizophrenia susceptibility genes identified by large-scale GWAS meta-analysis (Trubetskoy et al., 2022). Therefore, it appears that the genetic variants for AAO do not show very substantial overlap with the susceptibility variants for schizophrenia, although further and larger-scale studies on AAO are needed before any conclusion can be drawn. On the other hand, modifier genes refer to the genes that do not directly influence disease susceptibility, but affect the presentations of phenotypic features of a disease, such as symptom severity and AAO. The extant literature appears to support the ‘true modifier gene’ hypothesis for schizophrenia AAO. Lastly, susceptibility-modifier genes refer to genes that could cause a distinct form of a complex disease, and serve as “pseudo-modifier” genes. Such probable “pseudo-modifier” genes in the literature include dysbindin, *COMT*, and *DISC1* (Fanous and Kendler, 2005; DeRosse et al., 2006; Bergen, 2009). For example, dysbindin (*DTNBP1*) has been reported to be associated with schizophrenia in previous molecular studies (Pae et al., 2009; Wang et al., 2017). Interestingly, variants in *DTNBP1* was also reported to show associations with the severity of negative symptoms (Fanous et al., 2005; DeRosse et al., 2006). Some candidate-gene studies in this review may partially support the hypothesis (Dubertret et al., 2001; Numata et al., 2007; Voisey et al., 2012; Alfimova et al., 2023) that some genes may be “pseudo-modifiers” for AAO. More future research is needed to clarify these speculations.

Clinically, schizophrenia usually starts at the age of late teens and early 20s, yet less than 5% of schizophrenia patients would have AAO before the age of 18 (early-onset), and lesser still (around 1%–2%) before 13 (ultra-early-onset/childhood-onset) (Lin et al., 2016). In the literature, these adolescent- or childhood-onset schizophrenia patients were found to have more severe symptoms and poorer prognosis (Coulon et al., 2020). Classifying different schizophrenia subtypes based on AAO may enhance the biological homogeneity of more severe forms of schizophrenia. Similar to adult-onset schizophrenia, childhood-onset cases have strong familial aggregation characteristics, the early twin studies reported a concordance of 88.2% for monozygotic twins, which suggests that it is highly heritable (Kallmann and Roth, 1956). With the development of GWAS and sequencing technology, modern studies are able to search for specific genetic factors associated with the risk of schizophrenia more easily. Although GWAS of adult-onset schizophrenia has found nearly 300 common variants, these findings may not be directly generalizable to childhood-onset schizophrenia patients. Several challenges may hinder research on the genetics of childhood-onset schizophrenia. For example, the criteria of childhood-onset schizophrenia has not been well established, and the low prevalence of this severe form of schizophrenia (<1 in 10,000) has posed difficulty in subject recruitment (Bartlett, 2014), and previous research seldom precisely recorded AAO data (Burd and Kerbeshian, 1987). Another important direction is to clarify the role of other types of genetic variations, such as rare mutations, *de novo* variants and rare copy number variants (CNV) in early-onset, and childhood-onset schizophrenia, as well as AAO of the disorder (Fanous and Kendler, 2008; Loohuis et al., 2015; Trubetskoy et al., 2022).

A previous review argued that childhood-onset schizophrenia may share considerable genetic risk factors with adulthood-onset schizophrenia (Forsyth and Asarnow, 2020). However, childhood-onset schizophrenia may also carry a greater “cross-diagnosis genetic loading” of both common variants and CNVs that confer risk for other neurodevelopmental disorders such as ASD, learning disability and epilepsy (Kirov et al., 2014; Rees et al., 2016; Forsyth and Asarnow, 2020). Interestingly, two studies investigated early- and adulthood onset schizophrenia and calculated the PRS of these two groups, which suggested no significant associations between the PRS of schizophrenia and onset age (Bergen et al., 2014; Stepniak et al., 2014).

## Limitations

This review has several limitations. Although we conducted a search on relevant articles in three different databases, we did not quantitatively assess the issue of publication bias and perform quality assurance of retrieved studies. Having said that, this narrative review adheres to the SANRA guideline for reviews (Baethge et al., 2019). Moreover, we only provided qualitative descriptions of the retrieved studies, rather than pooling data together for analysis. Lastly, we did not compare the retrieved studies in a standardized manner.

## Future directions

Notwithstanding the aforementioned limitations, our results generally suggested that several strategies may be useful to better unveil the genetic architecture of AAO in schizophrenia patients. To address the power issue in GWAS, one strategy is to also consider gene-based or pathway-based association tests, which aggregates the effect of multiple genetic variants. Combining different cohorts is a useful way to increase sample size and thus increase the possibility of identifying putative variants. Nowadays, a growing number of large-scale GWAS meta-analysis have been performed. The findings from PGC GWAS meta-analyses on schizophrenia and other psychiatric disorders will enable researchers to explore the genetic basis of AAO using a greater variety of methods, such as computing polygenic risk scores based on different psychiatric phenotypes and testing their associations with AAO, and estimating genetic correlations across different phenotypes. On the other hand, findings from GWAS could only explain one-third to two-thirds of the heritability of complex traits (Tam et al., 2019), and 23% of the variation in liability to schizophrenia (Lee S. H. et al., 2012). Other types of research which study rare variants, gene expression analysis, and DNA methylation, will be useful in advancing our understanding of schizophrenia AAO.

To our knowledge, no study has adopted a multi-omics approach on AAO, and only a few studies have employed other omics approaches such as epigenomics and proteomics. One genome-wide methylation study has reported four intergenic CpG sites on the chromosome 2 that are associated with schizophrenia AAO (Srivastava et al., 2022). Another study reported that early-onset schizophrenia patients showed lower levels of global methylation (Wan and Wei, 2021). More research using these approaches is needed.

Some insights could be gained from epidemiological findings of schizophrenia AAO. First, gender difference in schizophrenia AAO has been consistently reported. Males have been found to have earlier AAO than females (Venkatesh et al., 2008; Eranti et al., 2013; Solmi et al., 2021), and females were found to have a second peak in their late 40–50 s, approximating the usual menopausal age (Li et al., 2016). This finding may implicate the roles of estrogen and related genetic variants, as well as the sex chromosome in influencing gender specific AAO. Consistent with the notion that female sex hormones may play a role in determining schizophrenia susceptibility or AAO, some previous studies reported a negative correlation between “age at menarche” and AAO in schizophrenia (Cohen et al., 1999; Kiliçaslan et al., 2014), yet such findings were not replicated in another study (Yazici et al., 2013). Importantly, few studies to date have investigated gene-environment interactions. For instance, early-onset schizophrenia has been reported to be highly associated with the cannabis use (Stepniak et al., 2014), and evidence suggested possible interaction between cannabis use and gender on AAO (Donoghue et al., 2014).

Lastly, it is noteworthy that the genetic architecture of AAO in other psychiatric disorders, such as bipolar disorder and major depressive disorder (MDD), have been relatively well-studied. For instance, a large-scale GWAS had combined 34 cohorts and investigated the genetic variants for determining AAO in bipolar disorder; they reported that one significant SNP rs1610275 was associated with earlier AAO. Other evidence suggested that the polygenic score (PGS) of autism spectrum disorder, MDD, schizophrenia, and educational attainment may contribute to earlier AAO in bipolar disorder (Kalman et al., 2021). More recently, another GWAS reported that early-onset type I bipolar disorder is significantly associated with the SNP rs11127876, located at the *CADM2* gene on chromosome 3, implicating its role in the central nervous system development (Wu et al., 2021). For MDD, an earlier study dissected the genetic heterogeneity of MDD based on AAO in a sample of >2,700 subjects, and the ‘clinical-case-only’ genome-wide analysis in AAO found 5 SNPs having reached the suggestive threshold of significance (Power et al., 2012). Another larger meta-analytic study utilized a cohort of 76,365 subjects with MDD in the UK Biobank, and reported that AAO was significantly heritable, with an estimated SNP-based heritability of 5.6% (Harder et al., 2022). Although this large GWAS meta-analysis (Harder et al., 2022) did not identify any SNPs or genes that reached SNP or gene-level significance, they found that AAO in MDD has genetic overlap with the risk for developing MDD (*r* = −0.49), as well as the risk of developing other psychiatric disorders, such as autism-spectrum disorder, schizophrenia, bipolar disorder, and anorexia nervosa. Taken together, patients with an earlier AAO of MDD or bipolar disorder may have a higher risk to develop other psychiatric disorders (Power et al., 2017; Harder et al., 2022).

## Conclusion

In summary, AAO is a very important phenotype of schizophrenia and a useful subtyping criterion, yet empirical evidence for genetic basis for AAO is currently limited and inconsistent. It remains unclear whether the genetic loci influencing AAO and the susceptibility genes for schizophrenia would overlap. Future research on the genetic basis of schizophrenia AAO would need refined conceptual models. This research topic has important clinical implications for advancing disease predictions and early interventions. The early psychosis intervention service has begun to orientate closer to child and adolescent psychiatry, which may eventually provide more clinical data on childhood- and adolescent-onset schizophrenia to facilitate genetic studies (Kelleher, 2023).
